# Concentration Changes in Plasma Amino Acids and Their Metabolites in Eventing Horses During Cross-Country Competitions

**DOI:** 10.3390/ani15131840

**Published:** 2025-06-22

**Authors:** Flora Philine Reemtsma, Johanna Giers, Stephanie Horstmann, Sabita Diana Stoeckle, Heidrun Gehlen

**Affiliations:** 1 Equine Clinic, Internal Medicine, Freie Universität Berlin, Oertzenweg 19b, 14193 Berlin, Germany; 2 Tierklinik Großmoor, Holzweg 13, 29352 Adelheidsdorf, Germany; 3 German Olympic Committee for Equestrian Sports (DOKR), Freiherr-von-Langen-Straße 15, 48231 Warendorf, Germany

**Keywords:** equine, amino acids, exercise, performance, athlete, fitness

## Abstract

Plasma amino acid (PAA) concentration in horses vary according to the exercise type. This study evaluated the changes in PAA levels and the associated metabolites, urea and ammonia, following short-duration, high-intensity cross-country exercise in eventing horses. Twenty eventing horses participated in 55 rides at 14 international competitions (2* to 4* levels) across five venues in Germany and Poland. Blood samples were collected at four timepoints: before exercise (TP0), at 10 min (TP1), and at 30 min (TP2) post-exercise, as well as in the morning on the day after the competition (TP3). A total of 23 different PAAs and two metabolites (ammonia and urea) were analyzed. PAA concentration difference over time was assessed by a mixed ANOVA. Significant fluctuations were observed in 18/25 parameters. For 21/23 PAAs, levels increased at TP1 and/or TP2, while cysteine concentrations decreased. Concentrations returned to pre-competition levels for 21/23 PAAs by TP3. Proline levels remained elevated (*p* = 0.002), while those of glycine significantly decreased (*p* = 0.027) at TP3. Plasma ammonia and urea levels increased at TP1, TP2 and TP3. This study provides foundations for supplementation strategies and can inform future works exploring PAAs’ role in performance and training adaptation in eventing horses and their potential as performance-related biomarkers.

## Simple Summary

Eventing horses are exposed to intense physical stress during the cross-country test. Amino acids play key roles in energy metabolism and muscle recovery. This study examines the changes in 23 plasma amino acid concentrations and two related metabolites (ammonia and urea) in twenty horses, before and after cross-country exercise at international competitions. Blood samples were obtained before, as well as at 10 and 30 min after exercise and on the next morning. Our results showed that plasma concentration levels for 21 out of the 23 amino acids examined increased after exercise, while cysteine levels decreased. Moreover, 21/23 plasma amino acid levels returned to baseline the next morning, while the concentration of proline stayed elevated, and that of glycine decreased. Ammonia and urea levels increased after exercise and remained elevated during the following morning. This study demonstrates that longitudinal changes in plasma amino acid concentrations occur at specific time points following cross country exercise in eventing horses.

## 1. Introduction

Amino acids (AAs) are fundamental molecular units that constitute proteins. These organic compounds primarily contain nitrogen within their amino groups (-NH_2_). Ammonia and urea are the most important molecules involved in protein metabolism and nitrogen excretion [1]. AAs play crucial roles in equine nutrition and physiology, protein synthesis, performance, and overall health [2,3,4]. Plasma concentrations are influenced by various factors including dietary intake [5,6,7,8,9,10], physical activity [11,12,13,14,15,16,17], as well as metabolic and health conditions, e.g., equine dysautonomia [18], septicemia [19], hepatic disease [20], pituitary pars intermedia dysfunction [21], equine metabolic syndrome, and insulin dysregulation [22].

During physical exercise, AA metabolism significantly affects skeletal muscle function [23]. Several studies have investigated the impact of exercise on plasma amino acid (PAA) and serum AA concentrations. Variability in exercise type (in terms of duration and intensity of the workout) and blood sampling time points have resulted in a wide range of reported changes in PAA concentrations, with various responses recorded in terms of PAA fluctuation across specific exercises. After long-lasting, low-intensity exercises such as 60 km or longer endurance rides, serum AA/PAA levels reportedly decrease [11,15,24]. In contrast, after 32 km endurance rides, increases in multiple measured AAs were found [11,24]. Investigations on changes in PAA levels during short-term and high-intensity exercise differ in their results. For instance, in standardbred trotters, plasma PAA concentrations increased within three minutes after exercise, while another study detected significant alterations in PAA profiles 60 min after standardized exercise test in standardbred horses: concentrations in 6/21 PAAs were increased, whereas 5/21 PAAs exhibited reduced concentrations [16]. Leucine and valine levels decrease immediately and within 30 min after jumping and reining exercises, whereas tryptophan levels increase [11]. Methionine and tyrosine levels were reduced 90 min post-moderate-intensity-exercise compared to 60 min post-exercise, whereas tryptophan demonstrated no time-dependent concentration differences [13]. Investigations of plasma ammonia levels showed an increase in plasma ammonia with incremental workload in horses [25] and humans [26]. Urea is known to be a sensitive marker for muscle fatigue in humans [27,28]. Urea levels increased in untrained individuals after mild exercise [29], as well as in endurance horses during an 80 km race [30] and in trained eventing horses after cross-country (CC) exercise [31], whereas these decreased in trained horses after mild treadmill exercise and showjumping competitions [29,32].

Eventing is a physically demanding equestrian sport comprising three sub-disciplines: dressage, CC, and jumping tests. In particular, CC is a high-intensity physical test that is reflected in significant physiological changes such as increased blood lactate levels and heart rate during and after competition [31,33]. To ensure optimal supplementation tailored to the specific demands of eventing horses, it is crucial to thoroughly analyze and understand the changes in PAA concentrations during CC exertion. Furthermore, it is important to determine which amino acids are consumed more rapidly and in greater quantities compared to others. Additionally, understanding the rate at which PAA concentrations recover post-exertion is essential for optimizing performance and recovery strategies. To the best of our knowledge, no prior studies have focused on changes in PAAs in eventing horses during and one day after a CC competition. The objective of this study was to examine alterations in PAA levels in high-performance eventing horses after CC exercise and to ascertain the time at which these values return to pre-exercise levels. We hypothesized that the PAA concentrations and the related metabolites, urea and ammonia, would increase following CC riding. Secondly, we hypothesized that the quantifiable increase in plasma concentrations would return to pre-exercise levels the morning after competition.

## 2. Materials and Methods

This study was registered and conducted as part of a research program investigating performance monitoring and cardiovascular health in eventing horses supervised by the German Olympic Committee for Equestrian Sports (DOKR). The previous results of this research program have been published in several studies [31,34,35,36]. Blood samples were drawn by veterinarians under the same conditions as in the two studies by Giers et al. and within the same pattern of sample collection [31,34]. The study requirements were fully explained to all participants, who were given the option to take part on a voluntary and unpaid basis. Written informed consent was obtained from all riders and horse owners. This study was registered with the Berlin State Office for Consumer Protection (registration number: 1-02.04.40.2022) VG006.

### 2.1. Horses

This longitudinal observational study involved horse–rider pairs who were enrolled in the “Performance Monitoring Program” of DOKR. This study was conducted on 20 horses from eight different riders. The horses ranged in age from 7 to 15 years with an average age of 11 years. The group included ten mares and ten geldings representing nine different warmblood breeds (Table 1). The horse identification details were retrieved from the FEI database [37]. The horses participated in CC competitions at 14 international eventing competitions held at five venues in Germany and Poland between March and September 2022 (calendar weeks 12–34), resulting in 55 starts.

### 2.2. Veterinary Check-Ups

Prior to the start of the study, a team of veterinarians conducted a comprehensive clinical, electrocardiographic, and echocardiographic examination of all equines. This process was performed to ensure the cardiovascular health of the participants. Before and after each competition, the horses underwent the required veterinary checks by the official FEI veterinarians and were confirmed to be “fit to compete.”

### 2.3. Training Schedules

Each horse was subjected to an individual training regimen devised by its rider and trainer to train the requisite skills to excel in a specific competition. The training routines incorporated a combination of dressage, jumping, CC exercises, and interval gallops. Furthermore, the schedules permitted periods of reduced activity and rest in the paddocks or pastures. However, the precise training regimen for each horse was not documented or subject to standardization.

### 2.4. Feeding

Horse feeding was not standardized and was individualized for each horse based on the personal preferences of the rider and trainer. The feeding regime was adapted to the activity and performance levels of the horses over the season. It contained forage and concentrate feed. The horses did not receive any type of amino acid supplements during the time of the study.

### 2.5. Competition Conditions

Horses participated in international eventing competitions from the two-star to the four-star level. Of the total of 55 rides, 9/55 rides (16.4%) were performed at the two-star level, 31/55 rides (56.4%) at the three-star level, and 15/55 rides (27.3%) at the four-star level. The mean length of the CC course was 3493.91 m, with a minimum length of 2661 m and a maximum length of 4455 m. Ridden speed depended on the level of competition but was 550 m/min on average. The ground was normal for 47/55 rides (85.5%) and deep for 8/55 of the total rides (14.5%). The ground profile was flat for 30/55 rides (54.5%), slightly hilly for 7/55 rides (12.7%), and hilly for 18/55 rides (32.7%). The weather on the competition days was sunny for 25/55 rides (45.5%) and cloudy for 30/55 rides (54.5%). The temperatures reached from 13 °C to 29 °C, with a mean temperature of 20 °C. All the competitions occurred in the temperate continental climatic zone of Middle Europe.

### 2.6. Performance During CC

A total of 51/55 (92.7%) CC rides were completed. In two of the four rides that were not completed, the horses and riders were eliminated from the course due to a fall from the horse at the end of the course. During the other two rides, the rider retired during the CC after two recorded refusals. The average penalty score in the CC competition was 9.16, ranging from 0 to 44 penalties. The mean placing after all three disciplines in the eventing competition was 14.18.

### 2.7. Blood Sampling

Blood samples were obtained at four defined time points: a baseline sample (TP0) collected in the morning before the exercise, post-exercise samples taken at 10 min (TP1) and 30 min (TP2) following completion of the CC exercise, and a final sample obtained the following morning between 4:00 AM and 7:30 AM (TP3) prior to concentrate feeding and any physical activity and 24 h (±1 h) after the TP0 sample collection. Due to the horses’ different starting times, the time interval between TP2 and TP3 ranged from 11 to 21 h.

Blood samples were collected by veterinarians as part of the DOKR Performance Monitoring Program. The blood collection site was disinfected with 1-propanol, and blood samples were collected from the jugular vein of the horses by a veterinarian using a Vacutainer system with 20 G needles and polyethylene terephthalate tubes. The EDTA blood tubes were centrifuged in a portable centrifuge (type EBA 200; Andreas Hettich GmbH & Co. KG (Tuttlingen, Germany)) at 1000× *g* for 10 min and within 10 min of sample collection. EDTA plasma for PAA analysis was transferred to uncoated plastic tubes and immediately stored at −20 °C. The frozen plasma samples were transported to MembraPure GmbH (Henningsdorf, Germany) for further analysis within 13 weeks after sample taking.

EDTA blood for lactate sampling was filled into 20 μL capillary tubes that were mixed with a lysing stabilization agent in a sample cup and stored at 5 °C immediately after sampling. Lactate samples were analyzed within 48 h of sample collection at the DOKR laboratory facilities using a BIOSEN system.

### 2.8. Amino Acid Measurements

Amino acid analysis was performed by MembraPure GmbH (Hennigsdorf, Germany). For the analysis, 400 µL EDTA plasma, 400 µL sample dilution buffer (including 100 nmol/mL norleucine standard), and 200 µL precipitation solution were added. The process of protein precipitation was conducted over a duration of 30 min, with the temperature maintained at 4 °C. The sample was then centrifuged at 14,100× *g* for 5 min. Amino acid concentrations were measured using the Aracus amino acid analyzer. The sample measurement sequence commenced with two standard measurements, followed by ten sample measurements. Subsequently, one standard measurement was performed for each set of ten samples. The sequences were determined using an additional standard measurement. All AAs and metabolites were measured at a wavelength of 570 nm, except for proline, which was measured at 440 nm.

### 2.9. Data Analysis

Statistical analyses were performed using SPSS (version 29.0.2.0 (20)). The Shapiro–Wilk test was used to assess the normal distribution of the data. Box plots were used to identify the outliers. Box tests for the equality of covariance matrices could not be performed. The Mauchly test was used to check the sphericity of each amino acid. In the absence of sphericity, the value of Greenhouse–Geisser ε was considered. If ε > 0.75, the Huynh–Feldt correction was applied. In cases where ε > 0.75 and ε < 0.75 occurred, the lower bound was evaluated. The data were not normally distributed and without outliers; however, as there was no non-parametric alternative to mixed ANOVA, mixed ANOVA was performed even if the assumptions were violated. The blood PAA concentration at each time point was used as the dependent variable. Individual horses (IDs) and rides were modeled with random intercepts. A separate mixed ANOVA was performed for each PAA. Differences in PAA concentrations at the time of measurement were determined using pairwise comparisons.

PAAs with 24.03% or more missing values were excluded from the study, as due to the high number of missing values, mixed ANOVA was not considered powerful. Bivariate Pearson correlation analysis was performed to analyze the linear relationship between lactate and PAA values.

### 2.10. Missing Samples

Owing to various circumstances (private reasons for the riders and complications related to the schedule of the event), 12 blood samples could not be collected at the tournament. These included three blood samples from TP1, seven from TP2, and two samples from TP3. Of the measured samples, 22 individual AA values could not be analyzed for technical reasons. For TP0, this included two missing values for glutamate, one missing value for histidine, one missing value for 1-methylhistidine, and one missing value for ammonia. At TP1, values were missing for alanine (2), asparagine (2), glutamate (1), cysteine (2), 1-methylhistidine (1), ornithine (1), and arginine (2). For TP3 alanine (1), glutamate (1), isoleucine (1), histidine (1), ornithine (1), and arginine (1) were missing. In total, 28 lactate values were missing because the TP0 and TP3 lactate levels were not measured at the beginning of the season, and one lactate value was missing at TP1.

## 3. Results

Aspartic acid and α-aminoadipic acid were excluded from the study owing to missing values, as their concentrations were below the detection level.

The mean PAA values, within-subject significance (omnibus *p*-value), and mean lactate values are shown in Table 2. Significant influences of the measured time point and the random intercepts ID and ride on PAA concentrations were observed for 18/25 measured parameters in our mixed model.

The lactate concentration values measured at TP1 varied considerably between the rides; more specifically, they displayed an approximate 26.08-unit difference with a minimum of 1.84 mmol/L and a maximum of 27.92 mmol/L. The inter-subject lactate concentration range at TP2 was 15.68, with minimum 0.8 mmol/L and maximum 16.48 mmol/L. Positive correlations (*p* < 0.05) were observed between lactate concentrations and 17 out of the 25 measured parameters. Our analysis indicated negative correlations between lactate and tryptophan (r = −0.171; *p* = 0.023), as well as cysteine (r = −0.259; *p* = 0.001). In contrast, no correlations were returned for lactate and 1-methylhistidine, histidine, glycine, asparagine, serine, and urea.

For 24/25 parameters, significant changes were observed in the concentrations quantified in the blood plasma at TP1 and/or TP2 compared to the initial value (TP0) (Table 3). Only asparagine demonstrated no statistically significant alterations in concentration before and after the exercise period and exhibited only a mild increase at TP2 (*p* = 0.129).

Following exercise, plasma concentrations for 22/25 parameters increased. A reduction in concentration was observed for cysteine. Tryptophan levels exhibited a decrease at TP1 (*p* = 0.023), with a subsequent increase in concentration during TP2 (*p* = 0.001). A marginal increase in plasma glycine values was recorded at TP2 (*p* = 0.031), followed by a decrease at TP3 (*p* = 0.027). Taurine, glutamate, isoleucine, leucine, ornithine, lysine, arginine, ammonia, and urea displayed the most rapid plasma level increases, with the most significant changes observed during TP1. By contrast, the concentrations of threonine, serine, glutamine, alanine, citrulline, valine, methionine, tyrosine, phenylalanine, histidine, 1-methylhistidine, and proline increased at a slower rate after exercise, with peak concentrations reached at TP2.

For the majority of PAA amounts no significant fluctuations between TP0 and TP3 were observed. Notably, ammonia (*p* = 0.008), urea (*p* = 0.046), and proline (*p* = 0.002) exhibited significantly higher concentrations at TP3 than at TP0. Nevertheless, the concentrations for 15 out of 23 PAAs (taurine, asparagine, threonine, serine, glutamine, citrulline, isoleucine, leucine, tyrosine, phenylalanine, histidine, tryptophan, ornithine, lysine, and arginine) at TP3 were slightly lower than their corresponding levels at TP0, but no significant differences were observed.

Increases in the mean values post-exercise to more than 150% from the base value were recorded for alanine (250.2% at TP2), taurine (189.4% at TP1), proline (155.6% at TP2), glutamate (151.9% at TP1), and ammonia (165.5% at TP1).

## 4. Discussion

To minimize muscle depletion, delay muscle fatigue, and enable equine athletes to perform their best, it is important to understand the exact processes involved in amino acid metabolism during exercise. This study demonstrated that the concentrations of free PAAs in eventing horses undergo alterations during CC riding. A total of 18/25 PAAs and metabolites showed significant changes at the measured time points.

The first hypothesis, suggesting that exercise increases PAA levels, was only partially confirmed; in this work, the levels of 21/23 PAAs and 2/2 metabolites increased, whereas cysteine and tryptophan showed a decline in concentration following exercise. Changes in PAA levels during exercise are primarily driven by muscle metabolism, as well as liver and kidney functions [14]. Increased PAA concentrations hours after exercise are caused by muscle efflux from exercise-induced protein degradation [38]. The patterns of concentration changes in free-circulating AAs in relation to exercise have been investigated in several previous studies, which have reported large differences in their alterations with different types of exercise. The CC test during an eventing competition is a unique type of physical work that, according to the authors, can be compared to short-term submaximal physical exercise. Our results are consistent with the observations in previous studies that investigated changes in blood AA levels during short-intensity exercise, such as following a standardized exercise test [14,16,38]. The analyses of a theoretical model of amino acid turnover in horses during exercise revealed that key limiting factors for anabolism are glutamine/glutamate, serine, and ornithine, while during high-intensity training, the demand for valine, lysine, histidine, and phenylalanine also increase, suggesting these eight AAs become limiting factors under conditions of severe physical exertion [39]. We found increased concentrations post-exercise in all mentioned PAAs.

Exercise-associated oxidative stress in horses remains a topic of ongoing discussions [34,40,41,42]. Several lines of evidence from the current body of literature in support of an exercise-related redox response include tryptophan, tyrosine, and methionine being regarded as antioxidant AAs because they are easily oxidized [43], with the decrease in cysteine levels after exercise being potentially part of an antioxidant response to the increased production of reactive oxygen species (ROS) following physical activity [44]. Cysteine is a key component of glutathione (GSH), a major antioxidant in muscle cells. During exercise, oxidative stress increases, and cysteine helps maintain the redox balance by contributing to the synthesis of GSH, which neutralizes ROS [45,46]. In a study performed in human cyclists, a 12% cysteine increase was observed after exercise [47]. However, consistent with our results, another study of overweight and normal-weight humans found decreased plasma cysteine levels immediately after exercise [44].

Tryptophan levels in our present work displayed an interesting changing pattern, with a decrease in TP1 and a subsequent increase in TP2. Increased tryptophan levels are associated with reduced aerobic performance [11,17,48]. The initial decrease in tryptophan levels that we observed is in line with findings from standardbred trotters and thoroughbreds following intense exercise [14,49] but has not been consistently reported [11,17]. While an increase in blood tryptophan can elevate brain serotonin levels and potentially lead to central fatigue, higher blood branched-chain amino acid (BCAA) levels compete with tryptophan for transport across the blood–brain barrier, thereby mitigating this effect [11,17,50,51]. A decrease in blood BCAA concentrations due to its uptake by active muscle as an energy source may result in an increased tryptophan/BCAA ratio [11,51]. BCAAs, including leucine, isoleucine, and valine, have garnered significant attention owing to their functions in exercise metabolism. Our results are consistent with those of other studies that reported elevated plasma concentrations of all BCAAs post-exercise [38,52]. Controversial reports exist in the literature regarding the importance of BCAAs in supplying energy during physical exercise. In equine studies, BCAAs appear to be preferentially utilized immediately after high-intensity exercise, as evidenced by the increased levels of their catabolic intermediates in skeletal muscle [23]. BCAAs contribute to energy production by providing intermediates for the tricarboxylic acid cycle [53]. Despite suggestions that BCAAs may serve as an additional fuel source during exercise alongside carbohydrates and fats, enzyme activities involved in their oxidation appear to be too low to make a major contribution to the overall energy expenditure; thus, AA metabolism is considered a minor energy source [54,55,56].

In this study, plasma lactate levels exhibited a mean concentration of 10.37 mmol/L at 10 min post-exercise, indicating the onset of anaerobic muscle metabolism during the CC exercise. Lactate is key indicator of anaerobic muscle activity, and its systemic levels increase significantly according to the intensity of exercise, indicating a shift towards anaerobic metabolism [31,57,58,59]. In ten rides, the lactate values exhibited only a minor increase following exercise (TP1 lactate < 5 mmol/L), indicating that not all rides were associated with submaximal exertion of the horses. In standardbred horses, lactate concentration after exercise significantly correlated with race performance [57]. We observed correlations between lactate and PAA concentrations in 16/23 PAAs and ammonia. Negative correlations were returned for lactate and cysteine (r = −0.259; *p* = 0.001), as well as tryptophan (r = −0.171; *p* = 0.023). This reflected the observed patterns in the measured parameters and confirmed the findings of another study which showed a negative correlation between lactate and tryptophan and a positive correlation between lactate and taurine [14].

As this study found no significant differences in the 21/23 measured PAA concentrations between TP0 and TP3, we partially accepted our second hypothesis that PAA levels return to pre-exercise baseline values within the next day. Proline, ammonia, and urea concentrations remained significantly elevated the next morning, whereas glycine concentration decreased. Ammonia is a product of the deamination of AA in the liver during protein breakdown and AA metabolism. In turn, urea is the main and less toxic byproduct of the urea cycle (ornithine cycle), which catabolizes ammonia via carbon dioxide and a series of enzymatic reactions in the liver [1]. Urea is then transported to the kidneys via the bloodstream for elimination. As these metabolic processes occur over longer periods after exercise, it is reasonable to expect a delayed recovery of these values. The evaluation of PAA levels showed that almost all TP3 values were slightly below the pre-exercise levels. This underscores the necessity for further research to elucidate the most effective methods of amino acid supplementation for eventing horses, as they are exposed to particular forms of physical stress during such 3-day events and are required to complete showjumping competitions the day after the CC test.

Previous studies have shown that supplementation can influence PAA levels. Enrichment of horse feed with a protein/AA supplement within one hour after exercise significantly elevated the intramuscular levels of specific AAs [38], which showcases the usefulness of supplementing targeted AAs to support anabolic mechanisms [38]. Another study demonstrated the rapid absorption of AA in the gut [7]. Feeding an essential AA mixture post-exercise was shown to partly decrease proteolysis [40].

Our study had several limitations, primarily regarding the feeding regime, which was not standardized owing to an inability of ensuring that the horses received consistent feed. Instead, the horses were fed according to their individual needs, and it was not possible to guarantee a standardized regime for their maintenance. However, in principle, horses are kept in boxes and have a regular free range in the form of paddocks or grazing. Despite the lack of a standardized feeding program in this study, the pre-exercise values mainly conformed with those seen in other studies on standardbred horses [14,16], as PAA concentrations were within the same ranges. However, these minor deviations do not affect the validity of the study. The pre-exercise values of ornithine, proline, glycine, valine, citrulline, isoleucine, and glutamate were lower in our study compared to the values reported by Westermann et al. [16] but aligned with those obtained by Hackl et al. [14]. We found taurine, glutamine, and arginine plasma levels to be lower in our study than their respective concentrations reported by Hackl et al. [14], whereas the values for lysine, valine, alanine, and threonine were only marginally higher in our work.

The non-standardized training protocols accounted for another notable limitation of the current study. Training was conducted at the discretion of the riders and trainers, with the horses being prepared for the performance level of the respective test, which was planned according to its severity.

A further limitation was the missing adjustment for plasma volume shift during exercise. The alterations in several blood parameters of horses, conduced in this study, have previously been reported by Giers et al. [31,34]. As the present study involved the same cohort of horses and employed the same exercise, the plasma volume shift of 10% calculated by Giers et al. [31,34] can be directly applied. Based on this assumption, our study’s findings remain valid, as only 4 out of the 25 parameters (tryptophan, serine, glycine, and citrulline) exhibited a difference of less than 10% post-exercise compared to the pre-exercise state.

Another limitation encountered was that the horses were examined for cardiovascular health at the beginning of the study but not for any other metabolic diseases. Nevertheless, all horses were able to compete in high-level international eventing competitions, which require good overall health. The TP0 lactate values at the beginning of the season were missing, leaving the lactate data incomplete. The subsequent morning values were measured at non-uniform times (11:00–21:00 h after TP2), as the respective schedules of the tests were individually designed by the organizer. However, we focused on a uniform time period between TP0 and TP3. Although the values were measured prior to the horses being moved and granted access to concentrate feed the next morning, the varying time intervals between the measurements could result in inaccurate recovery values.

Although we did not standardize the feeding and exercise regimens in this study, we were able to clearly assign PAA changes to the exercise impact. We showed that significant changes occurred in almost all measured PAA concentrations after a CC event owing to their consumption during physical work. Increases in mean values post-exercise to more than 150% were observed for alanine, taurine, proline, glutamate, and ammonia, which could potentially serve as performance-predictive parameters due to their marked alteration following exercise. Nevertheless, further research is required to evaluate the relationships between these parameters and individual performances.

Future studies should attempt to determine performance-predictive blood parameters (for example, pre-performance PAA values). Subsequent studies would need to focus on tracking individual changes over time to better assess the potential influence of training on PAA levels.

## 5. Conclusions

This study highlights the dynamic alterations in PAA concentrations and their metabolites in eventing horses after CC exercise. Significant changes were observed, with variations in peak concentrations at different time points post-exercise. Despite the identified limitations, such as non-standardized feeding regimens, the findings indicate that CC exercise significantly influences PAA levels. Future research should explore performance-predictive blood parameters and the role of PAAs in training adaptation to optimize equine athletic performance.

## Figures and Tables

**Table 1:** Age, breed, sex, and number of completed competitions in season 2022 of the study’s participating horses.

Horse	Age	Breed	Sex	Competitions During Season 2022
1	7	Holsteiner	Mare	4
2	7	Oldenburger	Mare	6
3	7	Hanoverian	Mare	6
4	8	Oldenburger	Mare	7
5	8	Westphalian	Gelding	4
6	9	Hanoverian	Gelding	7
7	9	German Sport Horse	Mare	4
8	10	Polish Horse Breeders Association	Mare	7
9	10	Irish Sport Horse	Gelding	4
10	11	Hanoverian	Gelding	5
11	11	Holsteiner	Gelding	6
12	12	Stud Book du Cheval Selle Francais	Mare	6
13	12	Hanoverian	Gelding	6
14	12	Irish Sport Horse	Gelding	6
15	12	Hanoverian	Mare	7
16	14	Hanoverian	Gelding	6
17	14	Holsteiner	Gelding	3
18	15	Hanoverian	Mare	5
19	15	Rheinlander	Mare	2
20	15	Hanoverian	Gelding	8

**Table 2:** Descriptive mean values (M) and the standard deviation (SD) of plasma amino acids, metabolites and lactate at the measured time points, with the corresponding omnibus *p*-value. A–D mark the considered method for within-subject effects; A—Assumed Sphericity, (B—Greenhouse–Geisser), C—Huynh–Feldt, and D—Lower Bound. Significant within-subjects effects (*p* < 0.05) are highlighted in bold.

	M (SD) TP0	M (SD) TP1	M (SD) TP2	M (SD) TP3	*p*
Alanine [µmol/L]	213.30 (59.70)	525.00 (143.86)	533.77 (138.03)	215.10 (70.18)	**<0.001** ^A^
Arginine [µmol/L]	91.88 (20.25)	105.38 (18.57)	105.49 (22.13)	84.47 (21.59)	0.051 ^D^
Asparagine [µmol/L]	34.47 (19.91)	33.30 (14.94)	40.26 (17.73)	34.98 (21.97)	0.140 ^A^
Citrulline [µmol/L]	74.61 (20.47)	81.59 (34.00)	81.28 (16.35)	70.76 (28.48)	0.120 ^D^
Cysteine [µmol/L]	17.99 (10.57)	11.73 (10.09)	14.31 (8.92)	18.89 (11.35)	**0.004** ^C^
Glutamine [µmol/L]	310.69 (90.23)	342.02 (94.66)	355.43 (103.88)	290.85 (94.84)	**<0.001** ^A^
Glutamate [µmol/L]	30.66 (22.57)	46.58 (19.10)	41.12 (17.79)	28.52 (15.88)	**0.033** ^D^
Glycine [µmol/L]	489.77 (120.30)	494.65 (106.87)	508.80 (105.75)	430.00 (114.03)	**<0.001** ^A^
Histidine [µmol/L]	78.44 (14.81)	79.71 (18.12)	89.36 (12.28)	75.98 (17.83)	0.077 ^D^
Isoleucine [µmol/L]	56.35 (13.58)	72.97 (19.61)	68.49 (16.74)	52.31 (17.64)	**0.020** ^A^
Leucine [µmol/L]	98.34 (23.86)	142.63 (27.33)	134.50 (36.98)	90.59 (30.35)	**<0.001** ^A^
Lysine [µmol/L]	95.93 (25.01)	122.80 (27.98)	122.22 (29.07)	86.36 (25.38)	**0.025** ^D^
Methionine [µmol/L]	29.24 (5.73)	37.75 (10.43)	41.09 (10.88)	28.78 (7.64)	**<0.001** ^A^
1-Methylhistidine [µmol/L]	19.56 (8.79)	22.31 (13.18)	22.03 (8.76)	20.66 (15.61)	0.145 ^D^
Ornithine [µmol/L]	59.30 (14.67)	74.44 (17.24)	69.70 (20.24)	54.79 (14.15)	0.017 ^D^
Phenylalanine [µmol/L]	62.73 (10.94)	82.94 (11.17)	84.81 (16.38)	59.15 (14.48)	**<0.001** ^A^
Proline [µmol/L]	113.19 (28.29)	144.30 (61.32)	176.18 (67.52)	125.96 (47.13)	0.455 ^D^
Serine [µmol/L]	246.14 (57.35)	250.56 (53.97)	258.23 (58.67)	229.82 (58.29)	**0.004** ^A^
Taurine [µmol/L]	31.45 (8.83)	59.65 (29,41)	58.25 (32.02)	29.20 (7.45)	**0.036** ^D^
Threonine [µmol/L]	134.21 (30.67)	157.24 (31.73)	171.46 (38.23)	125.01 (26.82)	**<0.001** ^A^
Tryptophan [µmol/L]	72.08 (13.88)	66.87 (11.80)	78.44 (12.70)	67.89 (14.45)	**<0.001** ^A^
Tyrosine [µmol/L]	62.05 (11.95)	75.84 (13.21)	85.77 (14.02)	58.59 (14.43)	**<0.001** ^A^
Valine [µmol/L]	186.79 (36.19)	216.45 (59.13)	227.77 (51.58)	179.92 (53.12)	**0.032** ^A^
Ammonia [µmol/L]	95.44 (44.28)	157.97 (70.35)	140.10 (64.66)	102.82 (45.78)	0.317 ^D^
Urea [µmol/L]	4836.50 (933.60)	5389.68 (842.86)	5415.54 (1072.58)	5144.07 (1088.35)	**0.003** ^A^
Lactate [mmol/L]	0.63 (0.26)	10.37 (6.14)	4.03 (3.13)	0.71 (0.31)	

**Table 3:** Statistically significant pairwise comparisons for the fluctuations in plasma concentration of each nitrogenous macromolecule between the different time points, calculated by mixed ANOVA. TP0 = pre-exercise, TP1 = 10 min post-exercise, TP2 = 30 min post-exercise, TP3 = next morning.

	*p* (TP0–TP1)	*p* (TP0–TP2)	*p* (TP0–TP3)	*p* (TP1–TP2)	*p* (TP1–TP3)	*p* (TP2–TP3)
Alanine	<0.001	<0.001	0.172	0.685	<0.001	<0.001
Arginine	<0.001	0.004	0.584	0.495	<0.001	0.010
Asparagine	0.700	0.129	0.780	0.066	0.509	0.423
Citrulline	0.100	0.001	0.968	0.781	0.004	0.146
Cysteine	<0.001	0.158	0.365	<0.001	<0.001	0.118
Glutamine	0.009	0.001	0.460	0.299	0.001	0.001
Glutamate	<0.001	0.012	0.758	0.001	<0.001	0.001
Glycine	0.370	0.031	0.027	0.273	0.005	<0.001
Histidine	0.863	<0.001	0.715	0.018	0.893	0.003
Isoleucine	<0.001	0.001	0.627	0.122	<0.001	0.004
Leucine	<0.001	<0.001	0.576	0.142	<0.001	<0.001
Lysine	<0.001	<0.001	0.172	0.762	<0.001	<0.001
Methionine	<0.001	<0.001	0.689	0.105	<0.001	<0.001
1-Methylhistidine	0.132	0.001	0.203	0.595	0.931	0.669
Ornithine	<0.001	0.007	0.569	0.115	<0.001	0.023
Phenylalanine	<0.001	<0.001	0.992	0.434	<0.001	<0.001
Proline	0.001	<0.001	0.002	<0.001	0.038	<0.001
Serine	0.204	0.026	0.605	0.266	0.069	0.022
Taurine	<0.001	<0.001	0.325	0.447	<0.001	<0.001
Threonine	<0.001	<0.001	0.153	0.005	<0.001	<0.001
Tryptophan	0.023	0.001	0.714	<0.001	0.193	0.008
Tyrosine	<0.001	<0.001	0.842	<0.001	<0.001	<0.001
Valine	0.012	<0.001	0.962	0.280	0.004	<0.001
Ammonia	<0.001	<0.001	0.008	0.282	<0.001	<0.001
Urea	<0.001	0.001	0.046	0.560	0.200	0.080

## Data Availability

The data presented in this study are available upon request from the corresponding author. These data are not publicly available because of privacy concerns.
